# Supplementary material for: Brain age has limited utility as a biomarker for capturing fluid cognition in older individuals
Source: eLife. 2024 Jun 13;12:RP87297. doi: 10.7554/eLife.87297 (PMC11175613; doi:10.7554/eLife.87297)
Supplement: Peer review history

# Editor report: eLife assessment

**DOI:** 10.7554/eLife.87297.4.sa0

**Authors:** Alex Fornito

**Affiliations:** Monash University Australia

**Keywords:** Solid, Useful

This **useful** manuscript challenges the utility of current paradigms for estimating brain-age with magnetic resonance imaging measures. It presents **solid** evidence to support the suggestion that an alternative approach focused on predicting cognition may be more beneficial. This work will be of interest to researchers working on brain-age and related models.

# Referee report: Reviewer #1 (Public Review)

**DOI:** 10.7554/eLife.87297.4.sa1

In this paper, the authors evaluate the utility of brain-age derived metrics for predicting cognitive decline by performing a 'commonality' analysis in a downstream regression that enables the different contribution of different predictors to be assessed. The main conclusion is that brain-age derived metrics do not explain much additional variation in cognition over and above what is already explained by age. The authors propose to use a regression model trained to predict cognition ('brain cognition') as an alternative suited to applications of cognitive decline. While this is less accurate overall than brain age, it explains more unique variance in the downstream regression.

Comments on revised version:

I thank the authors for the revision of the manuscript and for being more explicit about the inherent conceptual limitations of Brain Age / Brain Cognition. I have no further comments.

# Author comment: Author response

**DOI:** 10.7554/eLife.87297.4.sa2

**Authors:** Alina Tetereva, Narun Pat

**Affiliations:** University of Otago Dunedin New Zealand; University of Otago Dunedin New Zealand

The following is the authors’ response to the previous reviews.

**Reviewer #1 (Public Review):**

In this paper, the authors evaluate the utility of brain age derived metrics for predicting cognitive decline by performing a 'commonality' analysis in a downstream regression that enables the different contribution of different predictors to be assessed. The main conclusion is that brain age derived metrics do not explain much additional variation in cognition over and above what is already explained by age. The authors propose to use a regression model trained to predict cognition ('brain cognition') as an alternative suited to applications of cognitive decline. While this is less accurate overall than brain age, it explains more unique variance in the downstream regression.

Importantly, in this revision, we clarified that we did not intend to use Brain Cognition as an alternative approach. This is because, by design, the variation in fluid cognition explained by Brain Cognition should be higher or equal to that explained by Brain Age. Here we made this point more explicit and further stated that the relationship between Brain Cognition and fluid cognition indicates the upper limit of Brain Age’s capability in capturing fluid cognition. By examining what was captured by Brain Cognition, over and above Brain Age and chronological age via the unique effects of Brain Cognition, we were able to quantify the amount of co-variation between brain MRI and fluid cognition that was missed by Brain Age.

REVISED VERSION: while the authors have partially addressed my concerns, I do not feel they have addressed them all. I do not feel they have addressed the weight instability and concerns about the stacked regression models satisfactorily.

Please see our responses to Reviewer #1 Public Review #3 below

I also must say that I agree with Reviewer 3 about the limitations of the brain age and brain cognition methods conceptually. In particular that the regression model used to predict fluid cognition will by construction explain more variance in cognition than a brain age model that is trained to predict age. This suffers from the same problem the authors raise with brain age and would indeed disappear if the authors had a separate measure of cognition against which to validate and were then to regress this out as they do for age correction. I am aware that these conceptual problems are more widespread than this paper alone (in fact throughout the brain age literature), so I do not believe the authors should be penalised for that. However, I do think they can make these concerns more explicit and further tone down the comments they make about the utility of brain cognition. I have indicated the main considerations about these points in the recommendations section below.

Thank you so much for raising this point. We now have the following statement in the introduction and discussion to address this concern (see below).

Briefly, we made it explicit that, by design, the variation in fluid cognition explained by Brain Cognition should be higher or equal to that explained by Brain Age. That is, the relationship between Brain Cognition and fluid cognition indicates the upper limit of Brain Age’s capability in capturing fluid cognition. More importantly, by examining what was captured by Brain Cognition, over and above Brain Age and chronological age via the unique effects of Brain Cognition, we were able to quantify the amount of co-variation between brain MRI and fluid cognition that was missed by Brain Age. And this is the third goal of this present study.

From Introduction:

“Third and finally, certain variation in fluid cognition is related to brain MRI, but to what extent does Brain Age not capture this variation? To estimate the variation in fluid cognition that is related to the brain MRI, we could build prediction models that directly predict fluid cognition (i.e., as opposed to chronological age) from brain MRI data. Previous studies found reasonable predictive performances of these cognition-prediction models, built from certain MRI modalities (Dubois et al., 2018; Pat, Wang, Anney, et al., 2022; Rasero et al., 2021; Sripada et al., 2020; Tetereva et al., 2022; for review, see Vieira et al., 2022). Analogous to Brain Age, we called the predicted values from these cognition-prediction models, Brain Cognition. The strength of an out-of-sample relationship between Brain Cognition and fluid cognition reflects variation in fluid cognition that is related to the brain MRI and, therefore, indicates the upper limit of Brain Age’s capability in capturing fluid cognition. This is, by design, the variation in fluid cognition explained by Brain Cognition should be higher or equal to that explained by Brain Age. Consequently, if we included Brain Cognition, Brain Age and chronological age in the same model to explain fluid cognition, we would be able to examine the unique effects of Brain Cognition that explain fluid cognition beyond Brain Age and chronological age. These unique effects of Brain Cognition, in turn, would indicate the amount of co-variation between brain MRI and fluid cognition that is missed by Brain Age.”

From Discussion:

“Third, by introducing Brain Cognition, we showed the extent to which Brain Age indices were not able to capture the variation in fluid cognition that is related to brain MRI. More specifically, using Brain Cognition allowed us to gauge the variation in fluid cognition that is related to the brain MRI, and thereby, to estimate the upper limit of what Brain Age can do. Moreover, by examining what was captured by Brain Cognition, over and above Brain Age and chronological age via the unique effects of Brain Cognition, we were able to quantify the amount of co-variation between brain MRI and fluid cognition that was missed by Brain Age.

From our results, Brain Cognition, especially from certain cognition-prediction models such as the stacked models, has relatively good predictive performance, consistent with previous studies (Dubois et al., 2018; Pat, Wang, Anney, et al., 2022; Rasero et al., 2021; Sripada et al., 2020; Tetereva et al., 2022; for review, see Vieira et al., 2022). We then examined Brain Cognition using commonality analyses (Nimon et al., 2008) in multiple regression models having a Brain Age index, chronological age and Brain Cognition as regressors to explain fluid cognition. Similar to Brain Age indices, Brain Cognition exhibited large common effects with chronological age. But more importantly, unlike Brain Age indices, Brain Cognition showed large unique effects, up to around 11%. As explained above, the unique effects of Brain Cognition indicated the amount of co-variation between brain MRI and fluid cognition that was missed by a Brain Age index and chronological age. This missing amount was relatively high, considering that Brain Age and chronological age together explained around 32% of the total variation in fluid cognition. Accordingly, if a Brain Age index was used as a biomarker along with chronological age, we would have missed an opportunity to improve the performance of the model by around one-third of the variation explained.”

This is a reasonably good paper and the use of a commonality analysis is a nice contribution to understanding variance partitioning across different covariates. I have some comments that I believe the authors ought to address, which mostly relate to clarity and interpretation

Reviewer #1 Public Review #1

First, from a conceptual point of view, the authors focus exclusively on cognition as a downstream outcome. I would suggest the authors nuance their discussion to provide broader considerations of the utility of their method and on the limits of interpretation of brain age models more generally.

Thank you for your comments on this issue.

We now discussed the broader consideration in detail:

(1) the consistency between our findings on fluid cognition and other recent works on brain disorders,

(2) the difference between studies investigating the utility of Brain Age in explaining cognitive functioning, including ours and others (e.g., Butler et al., 2021; Cole, 2020, 2020; Jirsaraie, Kaufmann, et al., 2023) and those explaining neurological/psychological disorders (e.g., Bashyam et al., 2020; Rokicki et al., 2021)

and

(3) suggested solutions we and others made to optimise the utility of Brain Age for both cognitive functioning and brain disorders.

From Discussion:

“This discrepancy between the predictive performance of age-prediction models and the utility of Brain Age indices as a biomarker is consistent with recent findings (for review, see Jirsaraie, Gorelik, et al., 2023), both in the context of cognitive functioning (Jirsaraie, Kaufmann, et al., 2023) and neurological/psychological disorders (Bashyam et al., 2020; Rokicki et al., 2021). For instance, combining different MRI modalities into the prediction models, similar to our stacked models, ocen leads to the highest performance of age prediction models, but does not likely explain the highest variance across different phenotypes, including cognitive functioning and beyond (Jirsaraie, Gorelik, et al., 2023).”

“There is a notable difference between studies investigating the utility of Brain Age in explaining cognitive functioning, including ours and others (e.g., Butler et al., 2021; Cole, 2020, 2020; Jirsaraie, Kaufmann, et al., 2023) and those explaining neurological/psychological disorders (e.g., Bashyam et al., 2020; Rokicki et al., 2021). We consider the former as a normative type of study and the lader as a case-control type of study (Insel et al., 2010; Marquand et al., 2016). Those case-control Brain Age studies focusing on neurological/psychological disorders often build age-prediction models from MRI data of largely healthy participants (e.g., controls in a case-control design or large samples in a population-based design), apply the built age-prediction models to participants without vs. with neurological/psychological disorders and compare Brain Age indices between the two groups. On the one hand, this means that case-control studies treat Brain Age as a method to detect anomalies in the neurological/psychological group (Hahn et al., 2021). On the other hand, this also means that case-control studies have to ignore underfided models when applied prediction models built from largely healthy participants to participants with neurological/psychological disorders (i.e., Brain Age may predict chronological age well for the controls, but not for those with a disorder). On the contrary, our study and other normative studies focusing on cognitive functioning often build age prediction models from MRI data of largely healthy participants and apply the built age prediction models to participants who are also largely healthy. Accordingly, the age prediction models for explaining cognitive functioning in normative studies, while not allowing us to detect group-level anomalies, do not suffer from being under-fided. This unfortunately might limit the generalisability of our study into just the normative type of study. Future work is still needed to test the utility of brain age in the case-control case.”

“Next, researchers should not select age-prediction models based solely on age-prediction performance. Instead, researchers could select age-prediction models that explained phenotypes of interest the best. Here we selected age-prediction models based on a set of features (i.e., modalities) of brain MRI. This strategy was found effective not only for fluid cognition as we demonstrated here, but also for neurological and psychological disorders as shown elsewhere (Jirsaraie, Gorelik, et al., 2023; Rokicki et al., 2021). Rokicki and colleagues (2021), for instance, found that, while integrating across MRI modalities led to age prediction models with the highest age-prediction performance, using only T1 structural MRI gave age-prediction models that were better at classifying Alzheimer’s disease. Similarly, using only cerebral blood flow gave age-prediction models that were better at classifying mild/subjective cognitive impairment, schizophrenia and bipolar disorder.

As opposed to selecting age-prediction models based on a set of features, researchers could also select age-prediction models based on modelling methods. For instance, Jirsaraie and colleagues (2023) compared gradient tree boosting (GTB) and deep-learning brain network (DBN) algorithms in building age-prediction models. They found GTB to have higher age prediction performance but DBN to have better utility in explaining cognitive functioning. In this case, an algorithm with better utility (e.g., DBN) should be used for explaining a phenotype of interest. Similarly, Bashyam and colleagues (2020) built different DBN-based age-prediction models, varying in age-prediction performance. The DBN models with a higher number of epochs corresponded to higher age-prediction performance. However, DBN-based age-prediction models with a moderate (as opposed to higher or lower) number of epochs were better at classifying Alzheimer’s disease, mild cognitive impairment and schizophrenia. In this case, a model from the same algorithm with better utility (e.g., those DBN with a moderate epoch number) should be used for explaining a phenotype of interest.

Accordingly, this calls for a change in research practice, as recently pointed out by Jirasarie and colleagues (2023, p7), “Despite mounting evidence, there is a persisting assumption across several studies that the most accurate brain age models will have the most potential for detecting differences in a given phenotype of interest”. Future neuroimaging research should aim to build age-prediction models that are not necessarily good at predicting age, but at capturing phenotypes of interest.”

Reviewer #1 Public Review #2

Second, from a methods perspective, there is not a sufficient explanation of the methodological procedures in the current manuscript to fully understand how the stacked regression models were constructed. I would request that the authors provide more information to enable the reader to beUer understand the stacked regression models used to ensure that these models are not overfit.

Thank you for allowing us an opportunity to clarify our stacked model. We made additional clarification to make this clearer (see below). We wanted to confirm that we did not use test sets to build a stacked model in both lower and higher levels of the Elastic Net models. Test sets were there just for testing the performance of the models.

From Methods:

“We used nested cross-validation (CV) to build these prediction models (see Figure 7). We first split the data into five outer folds, leaving each outer fold with around 100 participants. This number of participants in each fold is to ensure the stability of the test performance across folds. In each outer-fold CV loop, one of the outer folds was treated as an outer-fold test set, and the rest was treated as an outer-fold training set. Ultimately, looping through the nested CV resulted in (a) prediction models from each of the 18 sets of features as well as (b) prediction models that drew information across different combinations of the 18 separate sets, known as “stacked models.” We specified eight stacked models: “All” (i.e., including all 18 sets of features), “All excluding Task FC”, “All excluding Task Contrast”, “Non-Task” (i.e., including only Rest FC and sMRI), “Resting and Task FC”, “Task Contrast and FC”, “Task Contrast” and “Task FC”. Accordingly, there were 26 prediction models in total for both Brain Age and Brain Cognition.

To create these 26 prediction models, we applied three steps for each outer-fold loop. The first step aimed at tuning prediction models for each of 18 sets of features. This step only involved the outer-fold training set and did not involve the outer-fold test set. Here, we divided the outer-fold training set into five inner folds and applied inner-fold CV to tune hyperparameters with grid search. Specifically, in each inner-fold CV, one of the inner folds was treated as an inner-fold validation set, and the rest was treated as an inner-fold training set. Within each inner-fold CV loop, we used the inner-fold training set to estimate parameters of the prediction model with a particular set of hyperparameters and applied the estimated model to the inner-fold validation set. Acer looping through the inner-fold CV, we, then, chose the prediction models that led to the highest performance, reflected by coefficient of determination (R2), on average across the inner-fold validation sets. This led to 18 tuned models, one for each of the 18 sets of features, for each outer fold.

The second step aimed at tuning stacked models. Same as the first step, the second step only involved the outer-fold training set and did not involve the outer-fold test set. Here, using the same outer-fold training set as the first step, we applied tuned models, created from the first step, one from each of the 18 sets of features, resulting in 18 predicted values for each participant. We, then, re-divided this outer-fold training set into new five inner folds. In each inner fold, we treated different combinations of the 18 predicted values from separate sets of features as features to predict the targets in separate “stacked” models. Same as the first step, in each inner-fold CV loop, we treated one out of five inner folds as an inner-fold validation set, and the rest as an inner-fold training set. Also as in the first step, we used the inner-fold training set to estimate parameters of the prediction model with a particular set of hyperparameters from our grid. We tuned the hyperparameters of stacked models using grid search by selecting the models with the highest R2 on average across the inner-fold validation sets. This led to eight tuned stacked models.

The third step aimed at testing the predictive performance of the 18 tuned prediction models from each of the set of features, built from the first step, and eight tuned stacked models, built from the second step. Unlike the first two steps, here we applied the already tuned models to the outer-fold test set. We started by applying the 18 tuned prediction models from each of the sets of features to each observation in the outer-fold test set, resulting in 18 predicted values. We then applied the tuned stacked models to these predicted values from separate sets of features, resulting in eight predicted values.

To demonstrate the predictive performance, we assessed the similarity between the observed values and the predicted values of each model across outer-fold test sets, using Pearson’s *r*, coefficient of determination (R2) and mean absolute error (MAE). Note that for R2, we used the sum of squares definition (i.e., R2 = 1 – (sum of squares residuals/total sum of squares)) per a previous recommendation (Poldrack et al., 2020). We considered the predicted values from the outer-fold test sets of models predicting age or fluid cognition, as Brain Age and Brain Cognition, respectively.”

**Author response image 1.** Diagram of the nested cross-validation used for creating predictions for models of each set of features as well as predictions for stacked models.

Note some previous research, including ours (Tetereva et al., 2022), splits the observations in the outer-fold training set into layer 1 and layer 2 and applies the first and second steps to layers 1 and 2, respectively. Here we decided against this approach and used the same outer-fold training set for both first and second steps in order to avoid potential bias toward the stacked models. This is because, when the data are split into two layers, predictive models built for each separate set of features only use the data from layer 1, while the stacked models use the data from both layers 1 and 2. In practice with large enough data, these two approaches might not differ much, as we demonstrated previously (Tetereva et al., 2022).

Reviewer #1 Public Review #3

Please also provide an indication of the different regression strengths that were estimated across the different models and cross-validation splits. Also, how stable were the weights across splits?

The focus of this article is on the predictions. Still, it is informative for readers to understand how stable the feature importance (i.e., Elastic Net coefficients) is. To demonstrate the stability of feature importance, we now examined the rank stability of feature importance using Spearman’s *ρ* (see Figure 4). Specifically, we correlated the feature importance between two prediction models of the same features, used in two different outer-fold test sets. Given that there were five outer-fold test sets, we computed 10 Spearman’s *ρ* for each prediction model of the same features. We found Spearman’s *ρ* to be varied dramatically in both age-prediction (*range*=.31-.94) and fluid cognition-prediction (*range*=.16-.84) models. This means that some prediction models were much more stable in their feature importance than others. This is probably due to various factors such as (a) the collinearity of features in the model, (b) the number of features (e.g., 71,631 features in functional connectivity, which were further reduced to 75 PCAs, as compared to 19 features in subcortical volume based on the ASEG atlas), (c) the penalisation of coefficients either with ‘Ridge’ or ‘Lasso’ methods, which resulted in reduction as a group of features or selection of a feature among correlated features, respectively, and (d) the predictive performance of the models. Understanding the stability of feature importance is beyond the scope of the current article. As mentioned by Reviewer 1, “The predictions can be stable when the coefficients are not,” and we chose to focus on the prediction in the current article.

**Author response image 2.** Stability of feature importance (i.e., Elastic Net Coefficients) of prediction models. Each dot represents rank stability (reflected by Spearman’s ρ) in the feature importance between two prediction models of the same features, used in two different outer-fold test sets. Given that there were five outer-fold test sets, there were 10 Spearman’s ρs for each prediction model. The numbers to the right of the plots indicate the mean of Spearman’s ρ for each prediction model.

Reviewer #1 Public Review #4

Please provide more details about the task designs, MRI processing procedures that were employed on this sample in addition to the regression methods and bias correction methods used. For example, there are several different parameterisations of the elastic net, please provide equations to describe the method used here so that readers can easily determine how the regularisation parameters should be interpreted.

Thank you for the opportunity for us to provide more methodical details.

First, for the task design, we included the following statements:

From Methods:

“HCP-A collected fMRI data from three tasks: Face Name (Sperling et al., 2001), Conditioned Approach Response Inhibition Task (CARIT) (Somerville et al., 2018) and VISual MOTOR (VISMOTOR) (Ances et al., 2009).

First, the Face Name task (Sperling et al., 2001) taps into episodic memory. The task had three blocks. In the encoding block [Encoding], participants were asked to memorise the names of faces shown. These faces were then shown again in the recall block [Recall] when the participants were asked if they could remember the names of the previously shown faces. There was also the distractor block [Distractor] occurring between the encoding and recall blocks. Here participants were distracted by a Go/NoGo task. We computed six contrasts for this Face Name task: [Encode], [Recall], [Distractor], [Encode vs. Distractor], [Recall vs. Distractor] and [Encode vs. Recall].

Second, the CARIT task (Somerville et al., 2018) was adapted from the classic Go/NoGo task and taps into inhibitory control. Participants were asked to press a budon to all [Go] but not to two [NoGo] shapes. We computed three contrasts for the CARIT task: [NoGo], [Go] and [NoGo vs. Go].

Third, the VISMOTOR task (Ances et al., 2009) was designed to test simple activation of the motor and visual cortices. Participants saw a checkerboard with a red square either on the lec or right. They needed to press a corresponding key to indicate the location of the red square. We computed just one contrast for the VISMOTOR task: [Vismotor], which indicates the presence of the checkerboard vs. baseline.”

Second, for MRI processing procedures, we included the following statements.

From Methods:

“HCP-A provides details of parameters for brain MRI elsewhere (Bookheimer et al., 2019; Harms et al., 2018). Here we used MRI data that were pre-processed by the HCP-A with recommended methods, including the MSMALL alignment (Glasser et al., 2016; Robinson et al., 2018) and ICA-FIX (Glasser et al., 2016) for functional MRI. We used multiple brain MRI modalities, covering task functional MRI (task fMRI), resting-state functional MRI (rsfMRI) and structural MRI (sMRI), and organised them into 19 sets of features.”

“Sets of Features 1-10: Task fMRI contrast (Task Contrast)

Task contrasts reflect fMRI activation relevant to events in each task. Bookheimer and colleagues (2019) provided detailed information about the fMRI in HCP-A. Here we focused on the pre-processed task fMRI Connectivity Informatics Technology Initiative (CIFTI) files with a suffix, “_PA_Atlas_MSMAll_hp0_clean.dtseries.nii.” These CIFTI files encompassed both the cortical mesh surface and subcortical volume (Glasser et al., 2013). Collected using the posterior-to-anterior (PA) phase, these files were aligned using MSMALL (Glasser et al., 2016; Robinson et al., 2018), linear detrended (see hdps://groups.google.com/a/humanconnectome.org/g/hcp-users/c/ZLJc092h980/m/GiihzQAUAwAJ) and cleaned from potential artifacts using ICA-FIX (Glasser et al., 2016).

To extract Task Contrasts, we regressed the fMRI time series on the convolved task events using a double-gamma canonical hemodynamic response function via FMRIB Software Library (FSL)’s FMRI Expert Analysis Tool (FEAT) (Woolrich et al., 2001). We kept FSL’s default high pass cutoff at 200s (i.e., .005 Hz). We then parcellated the contrast ‘cope’ files, using the Glasser atlas (Gordon et al., 2016) for cortical surface regions and the Freesurfer’s automatic segmentation (aseg) (Fischl et al., 2002) for subcortical regions. This resulted in 379 regions, whose number was, in turn, the number of features for each Task Contrast set of features. “

“Sets of Features 11-13: Task fMRI functional connectivity (Task FC)

Task FC reflects functional connectivity (FC ) among the brain regions during each task, which is considered an important source of individual differences (Elliod et al., 2019; Fair et al., 2007; Gradon et al., 2018). We used the same CIFTI file “_PA_Atlas_MSMAll_hp0_clean.dtseries.nii.” as the task contrasts. Unlike Task Contrasts, here we treated the double-gamma, convolved task events as regressors of no interest and focused on the residuals of the regression from each task (Fair et al., 2007). We computed these regressors on FSL, and regressed them in nilearn (Abraham et al., 2014). Following previous work on task FC (Elliod et al., 2019), we applied a highpass at .008 Hz. For parcellation, we used the same atlases as Task Contrast (Fischl et al., 2002; Glasser et al., 2016). We computed Pearson’s correlations of each pair of 379 regions, resulting in a table of 71,631 non-overlapping FC indices for each task. We then applied r-to-z transformation and principal component analysis (PCA) of 75 components (Rasero et al., 2021; Sripada et al., 2019, 2020). Note to avoid data leakage, we conducted the PCA on each training set and applied its definition to the corresponding test set. Accordingly, there were three sets of 75 features for Task FC, one for each task.

Set of Features 14: Resting-state functional MRI functional connectivity (Rest FC) Similar to Task FC, Rest FC reflects functional connectivity (FC ) among the brain regions, except that Rest FC occurred during the resting (as opposed to task-performing) period. HCPA collected Rest FC from four 6.42-min (488 frames) runs across two days, leading to 26-min long data (Harms et al., 2018). On each day, the study scanned two runs of Rest FC, starting with anterior-to-posterior (AP) and then with posterior-to-anterior (PA) phase encoding polarity. We used the “rfMRI_REST_Atlas_MSMAll_hp0_clean.dscalar.nii” file that was preprocessed and concatenated across the four runs. We applied the same computations (i.e., highpass filter, parcellation, Pearson’s correlations, r-to-z transformation and PCA) with the Task FC.

Sets of Features 15-18: Structural MRI (sMRI)

sMRI reflects individual differences in brain anatomy. The HCP-A used an established preprocessing pipeline for sMRI (Glasser et al., 2013). We focused on four sets of features: cortical thickness, cortical surface area, subcortical volume and total brain volume. For cortical thickness and cortical surface area, we used Destrieux’s atlas (Destrieux et al., 2010; Fischl, 2012) from FreeSurfer’s “aparc.stats” file, resulting in 148 regions for each set of features. For subcortical volume, we used the aseg atlas (Fischl et al., 2002) from FreeSurfer’s “aseg.stats” file, resulting in 19 regions. For total brain volume, we had five FreeSurfer-based features: “FS_IntraCranial_Vol” or estimated intra-cranial volume, “FS_TotCort_GM_Vol” or total cortical grey mader volume, “FS_Tot_WM_Vol” or total cortical white mader volume, “FS_SubCort_GM_Vol” or total subcortical grey mader volume and “FS_BrainSegVol_eTIV_Ratio” or ratio of brain segmentation volume to estimated total intracranial volume.”

Third, for regression methods and bias correction methods used, we included the following statements:

From Methods:

“For the machine learning algorithm, we used Elastic Net (Zou & Hastie, 2005). Elastic Net is a general form of penalised regressions (including Lasso and Ridge regression), allowing us to simultaneously draw information across different brain indices to predict one target variable. Penalised regressions are commonly used for building age-prediction models (Jirsaraie, Gorelik, et al., 2023). Previously we showed that the performance of Elastic Net in predicting cognitive abilities is on par, if not better than, many non-linear and morecomplicated algorithms (Pat, Wang, Bartonicek, et al., 2022; Tetereva et al., 2022). Moreover, Elastic Net coefficients are readily explainable, allowing us the ability to explain how our age-prediction and cognition-prediction models made the prediction from each brain feature (Molnar, 2019; Pat, Wang, Bartonicek, et al., 2022) (see below).

Elastic Net simultaneously minimises the weighted sum of the features’ coefficients. The degree of penalty to the sum of the feature’s coefficients is determined by a shrinkage hyperparameter ‘a’: the greater the a, the more the coefficients shrink, and the more regularised the model becomes. Elastic Net also includes another hyperparameter, ‘ℓ! ratio’, which determines the degree to which the sum of either the squared (known as ‘Ridge’; ℓ! ratio=0) or absolute (known as ‘Lasso’; ℓ! ratio=1) coefficients is penalised (Zou & Hastie, 2005). The objective function of Elastic Net as implemented by sklearn (Pedregosa et al., 2011) is defined as:

$$argmin_{\beta }\left(\frac{{\left|\left|y-X\right|\right|}_{2}^{2}}{2\times n_{samples}}+\alpha \times \ell_{1}\_ratio\times {\left|\left|\beta \right|\right|}_{1}+0.5\times \alpha \times \left(1-\ell_{1}\_ratio\right)\times {\left|\left|w\beta \right|\right|}_{2}^{2}\right),$$

where *X* is the features, *y* is the target, and b is the coefficient. In our grid search, we tuned two Elastic Net hyperparameters: a using 70 numbers in log space, ranging from .1 and 100, and ℓ!-ratio using 25 numbers in linear space, ranging from 0 and 1.

To understand how Elastic Net made a prediction based on different brain features, we examined the coefficients of the tuned model. Elastic Net coefficients can be considered as feature importance, such that more positive Elastic Net coefficients lead to more positive predicted values and, similarly, more negative Elastic Net coefficients lead to more negative predicted values (Molnar, 2019; Pat, Wang, Bartonicek, et al., 2022). While the magnitude of Elastic Net coefficients is regularised (thus making it difficult for us to interpret the magnitude itself directly), we could still indicate that a brain feature with a higher magnitude weights relatively stronger in making a prediction. Another benefit of Elastic Net as a penalised regression is that the coefficients are less susceptible to collinearity among features as they have already been regularised (Dormann et al., 2013; Pat, Wang, Bartonicek, et al., 2022).

Given that we used five-fold nested cross validation, different outer folds may have different degrees of ‘a’ and ‘ℓ! ratio’, making the final coefficients from different folds to be different. For instance, for certain sets of features, penalisation may not play a big part (i.e., higher or lower ‘a’ leads to similar predictive performance), resulting in different ‘a’ for different folds. To remedy this in the visualisation of Elastic Net feature importance, we refitted the Elastic Net model to the full dataset without spli{ng them into five folds and visualised the coefficients on brain images using Brainspace (Vos De Wael et al., 2020) and Nilern (Abraham et al., 2014) packages. Note, unlike other sets of features, Task FC and Rest FC were modelled acer data reduction via PCA. Thus, for Task FC and Rest FC, we, first, multiplied the absolute PCA scores (extracted from the ‘components_’ attribute of ‘sklearn.decomposition.PCA’) with Elastic Net coefficients and, then, summed the multiplied values across the 75 components, leaving 71,631 ROI-pair indices. *“*

References

Abraham, A., Pedregosa, F., Eickenberg, M., Gervais, P., Mueller, A., Kossaifi, J., Gramfort, A., Thirion, B., & Varoquaux, G. (2014). Machine learning for neuroimaging with scikitlearn. *Frontiers in Neuroinformatics*, *8*, 14. hdps://doi.org/10.3389/fninf.2014.00014

Ances, B. M., Liang, C. L., Leontiev, O., Perthen, J. E., Fleisher, A. S., Lansing, A. E., & Buxton, R. B. (2009). Effects of aging on cerebral blood flow, oxygen metabolism, and blood oxygenation level dependent responses to visual stimulation. *Human Brain Mapping*, *30*(4), 1120–1132. hdps://doi.org/10.1002/hbm.20574

Bashyam, V. M., Erus, G., Doshi, J., Habes, M., Nasrallah, I. M., Truelove-Hill, M., Srinivasan, D., Mamourian, L., Pomponio, R., Fan, Y., Launer, L. J., Masters, C. L., Maruff, P., Zhuo, C., Völzke, H., Johnson, S. C., Fripp, J., Koutsouleris, N., Saderthwaite, T. D., … on behalf of the ISTAGING Consortium, the P. A. disease C., ADNI, and CARDIA studies. (2020). MRI signatures of brain age and disease over the lifespan based on a deep brain network and 14 468 individuals worldwide. *Brain*, *143*(7), 2312–2324. hdps://doi.org/10.1093/brain/awaa160

Bookheimer, S. Y., Salat, D. H., Terpstra, M., Ances, B. M., Barch, D. M., Buckner, R. L., Burgess, G. C., Curtiss, S. W., Diaz-Santos, M., Elam, J. S., Fischl, B., Greve, D. N., Hagy, H. A., Harms, M. P., Hatch, O. M., Hedden, T., Hodge, C., Japardi, K. C., Kuhn, T. P., … Yacoub, E. (2019). The Lifespan Human Connectome Project in Aging: An overview. *NeuroImage*, *185*, 335–348. hdps://doi.org/10.1016/j.neuroimage.2018.10.009

Butler, E. R., Chen, A., Ramadan, R., Le, T. T., Ruparel, K., Moore, T. M., Saderthwaite, T. D., Zhang, F., Shou, H., Gur, R. C., Nichols, T. E., & Shinohara, R. T. (2021). Pi alls in brain age analyses. *Human Brain Mapping*, *42*(13), 4092–4101. hdps://doi.org/10.1002/hbm.25533

Cole, J. H. (2020). Multimodality neuroimaging brain-age in UK biobank: Relationship to biomedical, lifestyle, and cognitive factors. *Neurobiology of Aging*, *92*, 34–42. hdps://doi.org/10.1016/j.neurobiolaging.2020.03.014

Destrieux, C., Fischl, B., Dale, A., & Halgren, E. (2010). Automatic parcellation of human cortical gyri and sulci using standard anatomical nomenclature. *NeuroImage*, *53*(1), 1–15. hdps://doi.org/10.1016/j.neuroimage.2010.06.010

Dormann, C. F., Elith, J., Bacher, S., Buchmann, C., Carl, G., Carré, G., Marquéz, J. R. G., Gruber, B., Lafourcade, B., Leitão, P. J., Münkemüller, T., McClean, C., Osborne, P. E., Reineking, B., Schröder, B., Skidmore, A. K., Zurell, D., & Lautenbach, S. (2013). Collinearity: A review of methods to deal with it and a simulation study evaluating their performance. *Ecography*, *36*(1), 27–46. hdps://doi.org/10.1111/j.16000587.2012.07348.x

Dubois, J., Galdi, P., Paul, L. K., & Adolphs, R. (2018). A distributed brain network predicts general intelligence from resting-state human neuroimaging data. *Philosophical Transactions of the Royal Society B: Biological Sciences*, *373*(1756), 20170284. hdps://doi.org/10.1098/rstb.2017.0284

Elliod, M. L., Knodt, A. R., Cooke, M., Kim, M. J., Melzer, T. R., Keenan, R., Ireland, D., Ramrakha, S., Poulton, R., Caspi, A., Moffid, T. E., & Hariri, A. R. (2019). General functional connectivity: Shared features of resting-state and task fMRI drive reliable and heritable individual differences in functional brain networks. *NeuroImage*, *189*, 516–532. hdps://doi.org/10.1016/j.neuroimage.2019.01.068

Fair, D. A., Schlaggar, B. L., Cohen, A. L., Miezin, F. M., Dosenbach, N. U. F., Wenger, K. K., Fox, M. D., Snyder, A. Z., Raichle, M. E., & Petersen, S. E. (2007). A method for using blocked and event-related fMRI data to study “resting state” functional connectivity. *NeuroImage*, *35*(1), 396–405. hdps://doi.org/10.1016/j.neuroimage.2006.11.051

Fischl, B. (2012). FreeSurfer. *NeuroImage*, *62*(2), 774–781. hdps://doi.org/10.1016/j.neuroimage.2012.01.021

Fischl, B., Salat, D. H., Busa, E., Albert, M., Dieterich, M., Haselgrove, C., van der Kouwe, A., Killiany, R., Kennedy, D., Klaveness, S., Montillo, A., Makris, N., Rosen, B., & Dale, A. M. (2002). Whole Brain Segmentation. *Neuron*, *33*(3), 341–355. hdps://doi.org/10.1016/S0896-6273(02)00569-X

Glasser, M. F., Smith, S. M., Marcus, D. S., Andersson, J. L. R., Auerbach, E. J., Behrens, T. E. J., Coalson, T. S., Harms, M. P., Jenkinson, M., Moeller, S., Robinson, E. C., Sotiropoulos, S. N., Xu, J., Yacoub, E., Ugurbil, K., & Van Essen, D. C. (2016). The Human Connectome Project’s neuroimaging approach. *Nature Neuroscience*, *19*(9), 1175– 1187. hdps://doi.org/10.1038/nn.4361

Glasser, M. F., Sotiropoulos, S. N., Wilson, J. A., Coalson, T. S., Fischl, B., Andersson, J. L., Xu, J., Jbabdi, S., Webster, M., Polimeni, J. R., Van Essen, D. C., & Jenkinson, M. (2013). The minimal preprocessing pipelines for the Human Connectome Project. *NeuroImage*, *80*, 105–124. hdps://doi.org/10.1016/j.neuroimage.2013.04.127

Gordon, E. M., Laumann, T. O., Adeyemo, B., Huckins, J. F., Kelley, W. M., & Petersen, S. E. (2016). Generation and Evaluation of a Cortical Area Parcellation from Resting-State Correlations. *Cerebral Cortex*, *26*(1), 288–303. hdps://doi.org/10.1093/cercor/bhu239

Gradon, C., Laumann, T. O., Nielsen, A. N., Greene, D. J., Gordon, E. M., Gilmore, A. W., Nelson, S. M., Coalson, R. S., Snyder, A. Z., Schlaggar, B. L., Dosenbach, N. U. F., & Petersen, S. E. (2018). Functional Brain Networks Are Dominated by Stable Group and Individual Factors, Not Cognitive or Daily Variation. *Neuron*, *98*(2), 439-452.e5. hdps://doi.org/10.1016/j.neuron.2018.03.035

Hahn, T., Fisch, L., Ernsting, J., Winter, N. R., Leenings, R., Sarink, K., Emden, D., Kircher, T., Berger, K., & Dannlowski, U. (2021). From ‘loose fi{ng’ to high-performance, uncertainty-aware brain-age modelling. *Brain*, *144*(3), e31–e31. hdps://doi.org/10.1093/brain/awaa454

Harms, M. P., Somerville, L. H., Ances, B. M., Andersson, J., Barch, D. M., Bastiani, M., Bookheimer, S. Y., Brown, T. B., Buckner, R. L., Burgess, G. C., Coalson, T. S., Chappell, M. A., Dapredo, M., Douaud, G., Fischl, B., Glasser, M. F., Greve, D. N., Hodge, C., Jamison, K. W., … Yacoub, E. (2018). Extending the Human Connectome Project across ages: Imaging protocols for the Lifespan Development and Aging projects. *NeuroImage*, *183*, 972–984. hdps://doi.org/10.1016/j.neuroimage.2018.09.060

Insel, T., Cuthbert, B., Garvey, M., Heinssen, R., Pine, D. S., Quinn, K., Sanislow, C., & Wang, P. (2010). Research Domain Criteria (RDoC): Toward a New Classification Framework for Research on Mental Disorders. *American Journal of Psychiatry*, *167*(7), 748–751. hdps://doi.org/10.1176/appi.ajp.2010.09091379

Jirsaraie, R. J., Gorelik, A. J., Gatavins, M. M., Engemann, D. A., Bogdan, R., Barch, D. M., & Sotiras, A. (2023). A systematic review of multimodal brain age studies: Uncovering a divergence between model accuracy and utility. *PaUerns*, *4*(4), 100712. hdps://doi.org/10.1016/j.pader.2023.100712

Jirsaraie, R. J., Kaufmann, T., Bashyam, V., Erus, G., Luby, J. L., Westlye, L. T., Davatzikos, C., Barch, D. M., & Sotiras, A. (2023). Benchmarking the generalizability of brain age models: Challenges posed by scanner variance and prediction bias. *Human Brain Mapping*, *44*(3), 1118–1128. hdps://doi.org/10.1002/hbm.26144

Marquand, A. F., Rezek, I., Buitelaar, J., & Beckmann, C. F. (2016). Understanding Heterogeneity in Clinical Cohorts Using Normative Models: Beyond Case-Control Studies. *Biological Psychiatry*, *80*(7), 552–561. hdps://doi.org/10.1016/j.biopsych.2015.12.023

Molnar, C. (2019). *Interpretable Machine Learning. A Guide for Making Black Box Models Explainable*. hdps://christophm.github.io/interpretable-ml-book/

Nimon, K., Lewis, M., Kane, R., & Haynes, R. M. (2008). An R package to compute commonality coefficients in the multiple regression case: An introduction to the package and a practical example. *Behavior Research Methods*, *40*(2), 457–466. hdps://doi.org/10.3758/BRM.40.2.457

Pat, N., Wang, Y., Anney, R., Riglin, L., Thapar, A., & Stringaris, A. (2022). Longitudinally stable, brain-based predictive models mediate the relationships between childhood cognition and socio-demographic, psychological and genetic factors. *Human Brain Mapping*, hbm.26027. hdps://doi.org/10.1002/hbm.26027

Pat, N., Wang, Y., Bartonicek, A., Candia, J., & Stringaris, A. (2022). Explainable machine learning approach to predict and explain the relationship between task-based fMRI and individual differences in cognition. *Cerebral Cortex*, bhac235. hdps://doi.org/10.1093/cercor/bhac235

Pedregosa, F., Varoquaux, G., Gramfort, A., Michel, V., Thirion, B., Grisel, O., Blondel, M., Predenhofer, P., Weiss, R., Dubourg, V., Vanderplas, J., Passos, A., Cournapeau, D., Brucher, M., Perrot, M., & Duchesnay, É. (2011). Scikit-learn: Machine Learning in Python. *Journal of Machine Learning Research*, *12*(85), 2825–2830.

Poldrack, R. A., Huckins, G., & Varoquaux, G. (2020). Establishment of Best Practices for Evidence for Prediction: A Review. *JAMA Psychiatry*, *77*(5), 534–540. hdps://doi.org/10.1001/jamapsychiatry.2019.3671

Rasero, J., Sentis, A. I., Yeh, F.-C., & Verstynen, T. (2021). Integrating across neuroimaging modalities boosts prediction accuracy of cognitive ability. *PLOS Computational Biology*, *17*(3), e1008347. hdps://doi.org/10.1371/journal.pcbi.1008347

Robinson, E. C., Garcia, K., Glasser, M. F., Chen, Z., Coalson, T. S., Makropoulos, A., Bozek, J., Wright, R., Schuh, A., Webster, M., Huder, J., Price, A., Cordero Grande, L., Hughes, E., Tusor, N., Bayly, P. V., Van Essen, D. C., Smith, S. M., Edwards, A. D., … Rueckert, D. (2018). Multimodal surface matching with higher-order smoothness constraints. *NeuroImage*, *167*, 453–465. hdps://doi.org/10.1016/j.neuroimage.2017.10.037

Rokicki, J., Wolfers, T., Nordhøy, W., Tesli, N., Quintana, D. S., Alnæs, D., Richard, G., de Lange, A.-M. G., Lund, M. J., Norbom, L., Agartz, I., Melle, I., Nærland, T., Selbæk, G., Persson, K., Nordvik, J. E., Schwarz, E., Andreassen, O. A., Kaufmann, T., & Westlye, L. T. (2021). Multimodal imaging improves brain age prediction and reveals distinct abnormalities in patients with psychiatric and neurological disorders. *Human Brain Mapping*, *42*(6), 1714–1726. hdps://doi.org/10.1002/hbm.25323

Somerville, L. H., Bookheimer, S. Y., Buckner, R. L., Burgess, G. C., Curtiss, S. W., Dapredo, M., Elam, J. S., Gaffrey, M. S., Harms, M. P., Hodge, C., Kandala, S., Kastman, E. K., Nichols, T. E., Schlaggar, B. L., Smith, S. M., Thomas, K. M., Yacoub, E., Van Essen, D. C., & Barch, D. M. (2018). The Lifespan Human Connectome Project in Development: A large-scale study of brain connectivity development in 5–21 year olds. *NeuroImage*, *183*, 456–468. hdps://doi.org/10.1016/j.neuroimage.2018.08.050

Sperling, R. A., Bates, J. F., Cocchiarella, A. J., Schacter, D. L., Rosen, B. R., & Albert, M. S. (2001). Encoding novel face-name associations: A functional MRI study. *Human Brain Mapping*, *14*(3), 129–139. hdps://doi.org/10.1002/hbm.1047

Sripada, C., Angstadt, M., Rutherford, S., Kessler, D., Kim, Y., Yee, M., & Levina, E. (2019). Basic Units of Inter-Individual Variation in Resting State Connectomes. *Scientific Reports*, *9*(1), Article 1. hdps://doi.org/10.1038/s41598-018-38406-5

Sripada, C., Angstadt, M., Rutherford, S., Taxali, A., & Shedden, K. (2020). Toward a “treadmill test” for cognition: Improved prediction of general cognitive ability from the task activated brain. *Human Brain Mapping*, *41*(12), 3186–3197. hdps://doi.org/10.1002/hbm.25007

Tetereva, A., Li, J., Deng, J. D., Stringaris, A., & Pat, N. (2022). Capturing brain-cognition relationship: Integrating task-based fMRI across tasks markedly boosts prediction and test-retest reliability. *NeuroImage*, *263*, 119588. hdps://doi.org/10.1016/j.neuroimage.2022.119588

Vieira, B. H., Pamplona, G. S. P., Fachinello, K., Silva, A. K., Foss, M. P., & Salmon, C. E. G. (2022). On the prediction of human intelligence from neuroimaging: A systematic review of methods and reporting. *Intelligence*, *93*, 101654. hdps://doi.org/10.1016/j.intell.2022.101654

Vos De Wael, R., Benkarim, O., Paquola, C., Lariviere, S., Royer, J., Tavakol, S., Xu, T., Hong, S.J., Langs, G., Valk, S., Misic, B., Milham, M., Margulies, D., Smallwood, J., & Bernhardt, B. C. (2020). BrainSpace: A toolbox for the analysis of macroscale gradients in neuroimaging and connectomics datasets. *Communications Biology*, *3*(1), 103. hdps://doi.org/10.1038/s42003-020-0794-7

Woolrich, M. W., Ripley, B. D., Brady, M., & Smith, S. M. (2001). Temporal Autocorrelation in Univariate Linear Modeling of FMRI Data. *NeuroImage*, *14*(6), 1370–1386. hdps://doi.org/10.1006/nimg.2001.0931

Zou, H., & Hastie, T. (2005). Regularization and variable selection via the elastic net. *Journal of the Royal Statistical Society: Series B (Statistical Methodology)*, *67*(2), 301–320. hdps://doi.org/10.1111/j.1467-9868.2005.00503.x
